# Supplementary material for: Fracture toughness of a metal–organic framework glass
Source: Nat Commun. 2020 May 22;11:2593. doi: 10.1038/s41467-020-16382-7 (PMC7244719; doi:10.1038/s41467-020-16382-7)
Supplement: Supplementary file 1 — Supplementary Information [file 41467_2020_16382_MOESM1_ESM.pdf]

Supplementary Information for "Fracture toughness of a metal-organic framework glass" by To *et al*.

Fracture toughness of a metal-organic framework glass

Theany To[1,†], Søren S. Sørensen[1,†], Malwina Stepniewska[1], Ang Qiao[1], Lars R. Jensen[2], Mathieu Bauchy[3], Yuanzheng Yue[1], Morten M. Smedskjaer[1,*]

[1] *Department of Chemistry and Bioscience, Aalborg University, DK-9220, Aalborg, Denmark*

[2] *Department of Materials and Production, Aalborg University, DK-9220, Aalborg, Denmark*

[3] *Department of Civil and Environmental Engineering, University of California, Los Angeles, California 90095, USA*

[†] These authors contributed equally.

[*] Corresponding author. E-mail: mos@bio.aau.dk

Supplementary Tables

Supplementary Table 1. Comparison of fracture toughness (K_{Ic}) values of window glass obtained using the adapted and standard single edge precrack beam (SEPB) method. *S*, *W* and *B* are span length, specimen height and broadness, respectively. Source data are provided as a Source Data file.

**Method**	**Adapted SEPB (this study)**	**Standard SEPB (Ref[1])**
(*S*, *W* , *B*) [mm, mm, mm]	(7.5, 1.9, 1.5)	(20, 4, 3)
S/W [-]	~4	5
W/B [-]	1.27	1.33
K_{Ic} [MPa $m^{0.5}$]	0.72 ± 0.02	0.70 ± 0.01

Supplementary Figures

Supplementary Figure 1. Eight molecular dynamics simulated stress(σ)-strain(ε) curves starting from a relaxed simulation box that is subjected to tensile strain until fracture to compute the ultimate strength of ZIF-62 glass. Source data are provided as a Source Data file.

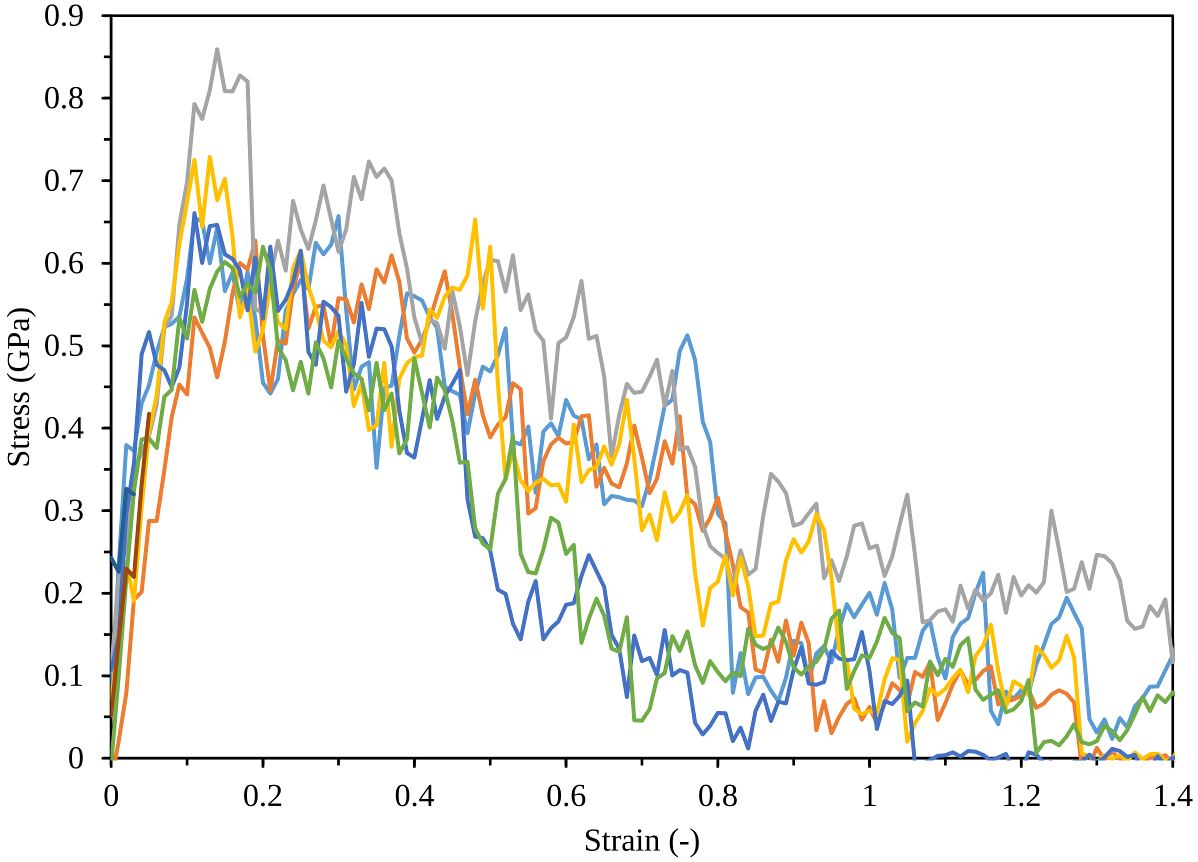

Supplementary Figure 2. Structural representation of an induced precrack in the molecular dynamics simulated ZIF-62 glass network after relaxation. Coloured spheres represent carbon (red), hydrogen (grey), nitrogen (green), and zinc (blue).

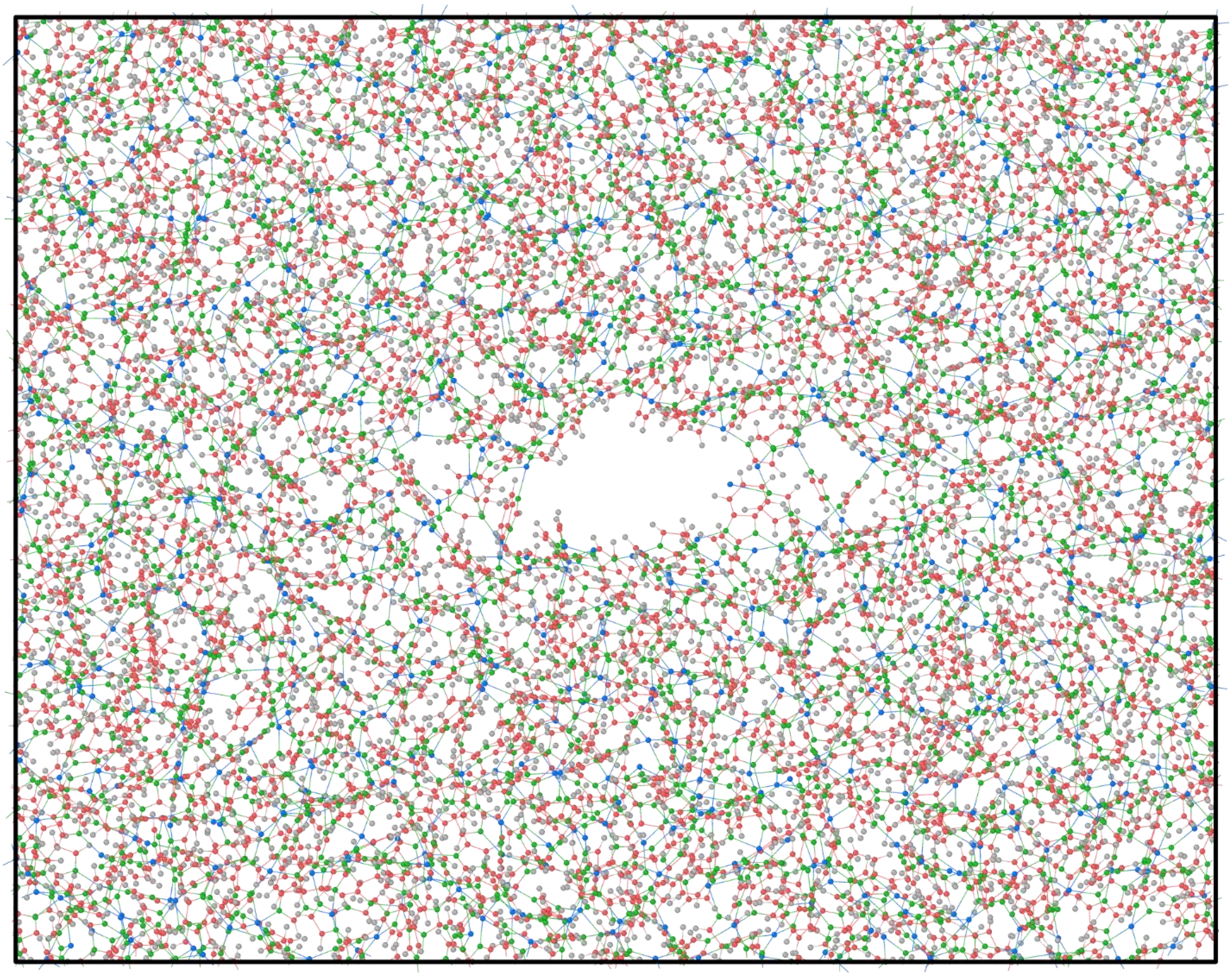

Supplementary Figure 3. Eight molecular dynamics simulated stress-strain curves of the precracked ZIF-62 glass system used in the estimation of its fracture toughness. Source data are provided as a Source Data file.

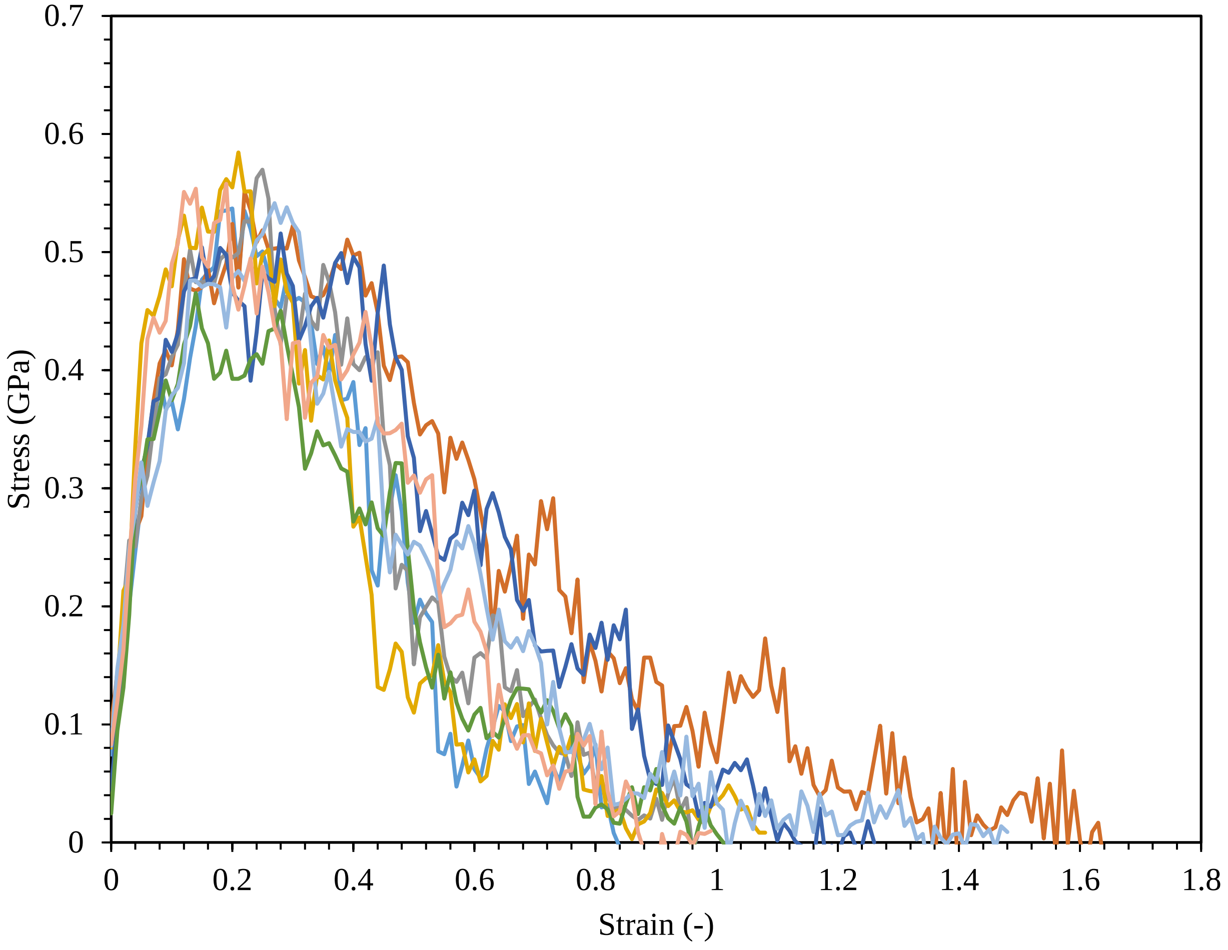

Supplementary Figure 4. Crack size dependence of the molecular dynamics simulated fracture toughness (K_{Ic}). By estimating K_{Ic} of a similar structure with three different crack sizes, we find its size dependence to be small and not systematic. The crack size of 36 Å is the valued used in the estimation of K_{Ic} in the main text. Source data are provided as a Source Data file.

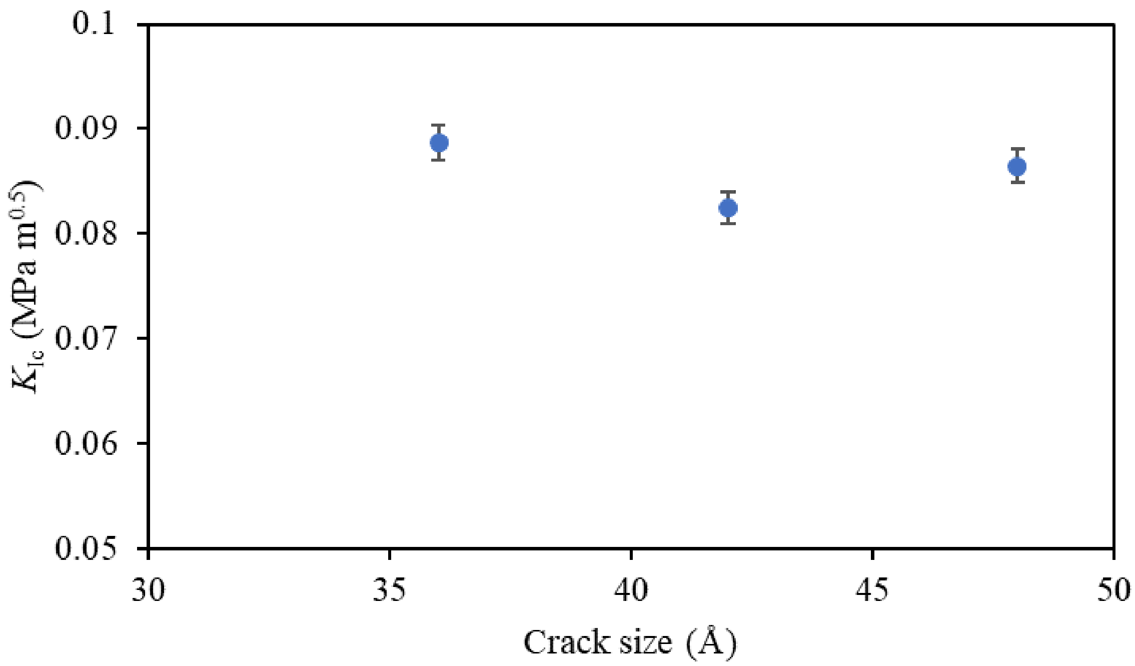

Supplementary Figure 5. Bond counting during the molecular dynamics simulated fracture process. **a** Total number of bonds during fracture. **b** Number of broken Zn-N bonds during fracture. **c** Total number of Zn-N bonds during fracture. In general, it is noticed how the amount of bonds increase during the fracture process. We ascribe this to structural rearrangements induced by the applied stress. Furthermore, we find that only Zn-N bonds are breaking and restructuring, as also seen from the similar shapes of the curves in a and c. Source data are provided as a Source Data file.

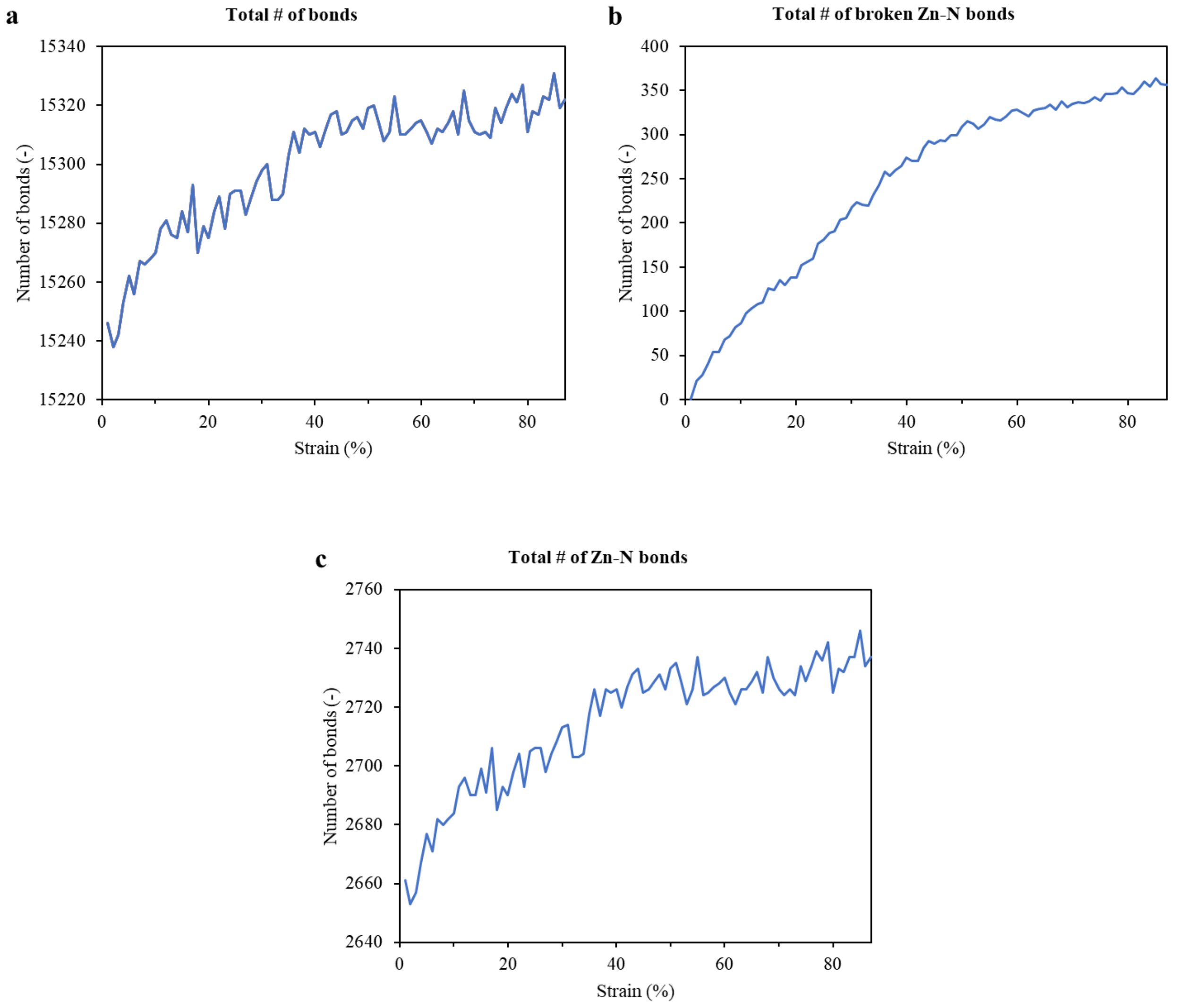

Supplementary Figure 6. Average normal stress as a function of strain during fracture. **a** Average normal stress of the *xx*, *yy*, and *zz* directions during fracture for each elemental species. **b** Average normal stress in the *xx* direction. **c** Average normal stress in the *yy* direction. **d** Average normal stress in the *zz* direction. All stresses are averages of eight structures during fracture. We have removed outliers in the simulations, which are believed to be caused by temporary compressive stresses appearing directly after severe bond ruptures (these data have not been sorted out in Supplementary Fig. 7). Interestingly, we find that only zinc atoms feature tensile stress in all normal directions, while carbon and nitrogen atoms feature normal stress varying between tensile and compressive depending on direction. Source data are provided as a Source Data file.

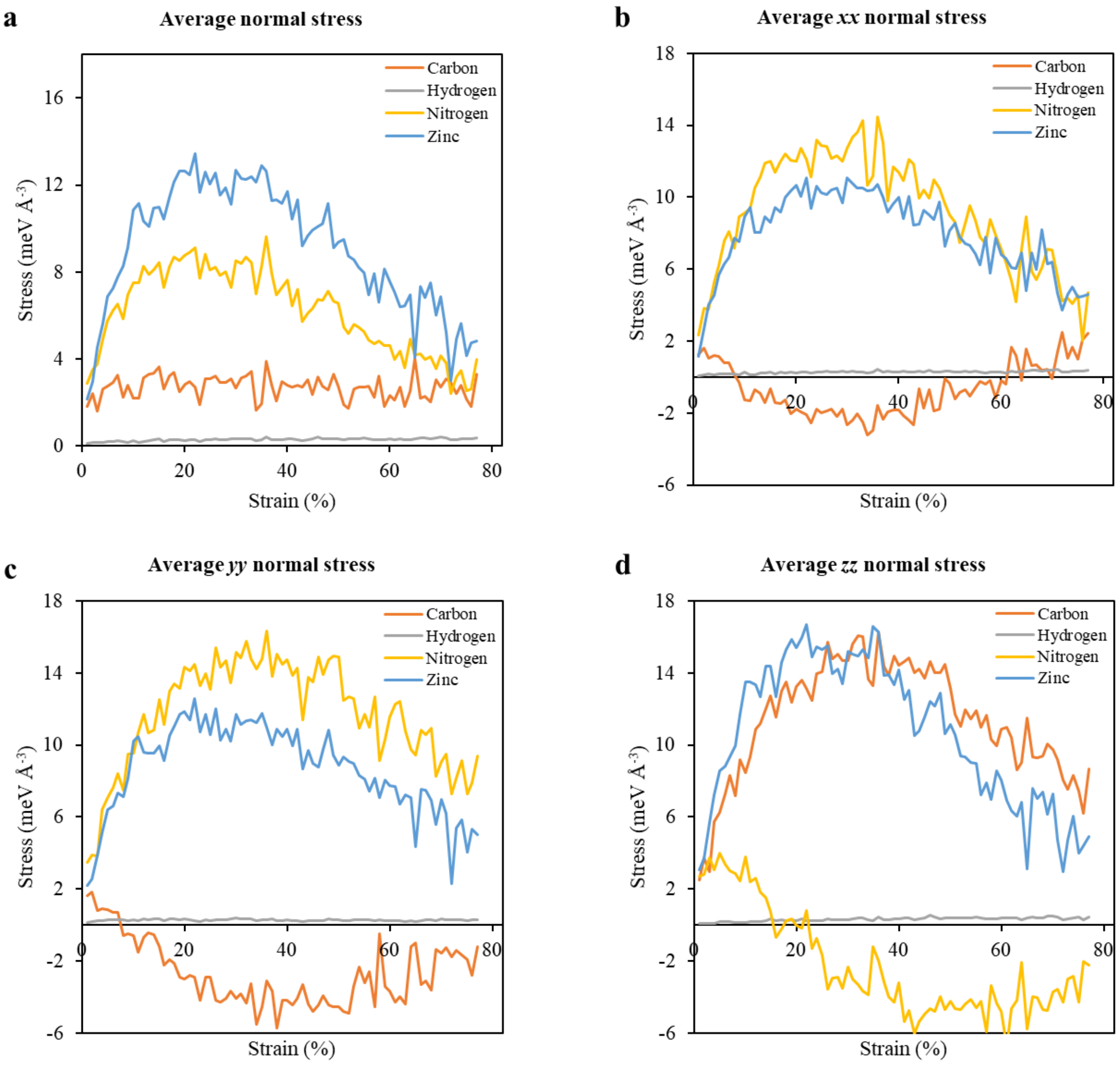

Supplementary Figure 7. Simulated summed distribution of stress of each elemental species for all normal directions with an induced pre-crack up until 82% strain. The results are shown for all eight simulated structures. **a** Carbon. **b** Hydrogen. **c** Nitrogen. **d** Zinc. We find stress to be exclusively distributed on carbon, nitrogen, and zinc atoms, while hydrogen atoms have nearly constant stress during fracture. Note that strain and stress axes are similar for all plots, but that the color reference scale differs between elements. Source data are provided as a Source Data file.

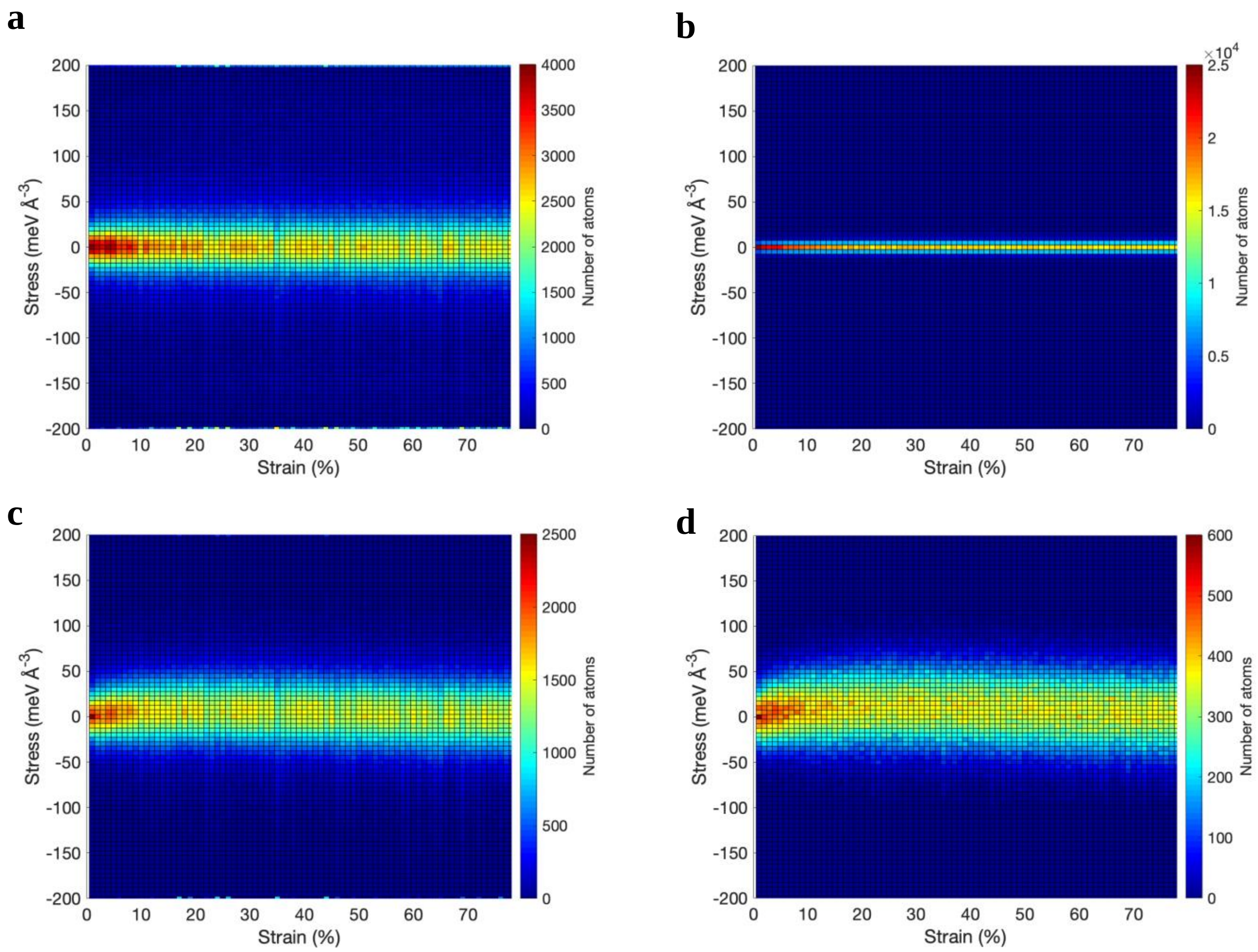

Supplementary Figure 8. XRD patterns of: as-synthesized crystalline ZIF-62 (black line), simulated ZIF-62 (orange line),[2] and bulk sample obtained by melt-quenching (blue line). By comparing simulated and experimental patterns, the as-synthesized powder is confirmed to be ZIF-62. The bulk glass sample shows the typical amorphous hump and no sharp crystalline peaks, confirming the amorphous nature of the sample. Source data are provided as a Source Data file.

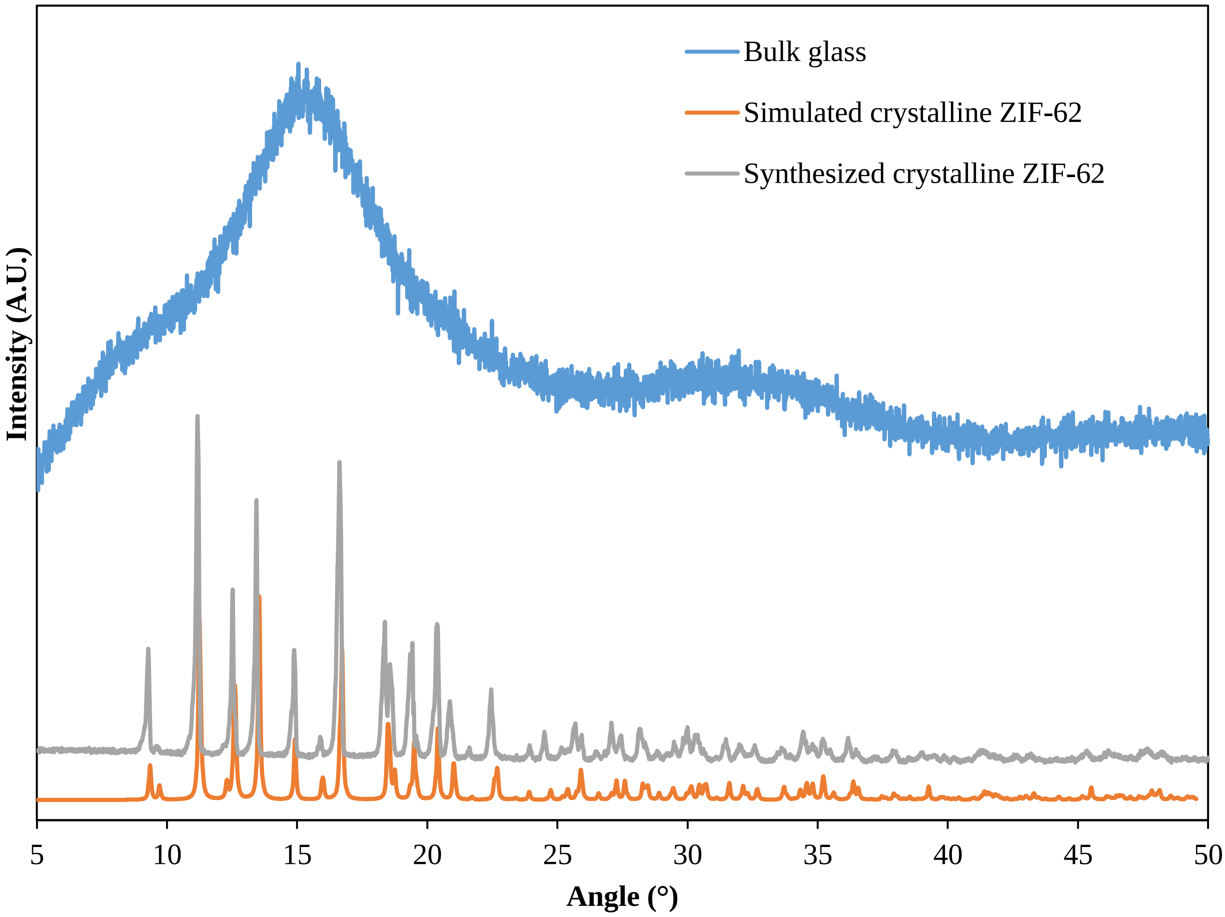

Supplementary References

1. To, T. *et al.* Fracture toughness, fracture energy and slow crack growth of glass as investigated by the Single-Edge Precracked Beam (SEPB) and Chevron-Notched Beam (CNB) methods. *Acta Mater.* **146**, 1–11 (2018).

2. Banerjee, R. *et al.* High-throughput synthesis of zeolitic imidazolate frameworks and application to CO_2 capture. *Science (80-.).* **319**, 939–943 (2008).
